# Measuring the Digital Competence of Health Professionals: Scoping Review

**DOI:** 10.2196/55737

**Published:** 2024-03-29

**Authors:** Anne Mainz, Julia Nitsche, Vera Weirauch, Sven Meister

**Affiliations:** 1 Health Informatics Faculty of Health, School of Medicine Witten/Herdecke University Witten Germany; 2 Department of Didactics and Educational Research in Health Science Faculty of Health, School of Medicine Witten/Herdecke University Witten Germany; 3 Department Healthcare Fraunhofer Institute for Software and Systems Engineering Dortmund Germany

**Keywords:** digital competence, digital literacy, digital health, health care, health care professional, health care professionals, scoping review

## Abstract

**Background:**

Digital competence is listed as one of the key competences for lifelong learning and is increasing in importance not only in private life but also in professional life. There is consensus within the health care sector that digital competence (or digital literacy) is needed in various professional fields. However, it is still unclear what exactly the digital competence of health professionals should include and how it can be measured.

**Objective:**

This scoping review aims to provide an overview of the common definitions of digital literacy in scientific literature in the field of health care and the existing measurement instruments.

**Methods:**

Peer-reviewed scientific papers from the last 10 years (2013-2023) in English or German that deal with the digital competence of health care workers in both outpatient and inpatient care were included. The databases ScienceDirect, Scopus, PubMed, EBSCOhost, MEDLINE, OpenAIRE, ERIC, OAIster, Cochrane Library, CAMbase, APA PsycNet, and Psyndex were searched for literature. The review follows the JBI methodology for scoping reviews, and the description of the results is based on the PRISMA-ScR (Preferred Reporting Items for Systematic Reviews and Meta-Analyses extension for Scoping Reviews) checklist.

**Results:**

The initial search identified 1682 papers, of which 46 (2.73%) were included in the synthesis. The review results show that there is a strong focus on technical skills and knowledge with regard to both the definitions of *digital competence* and the measurement tools. A wide range of competences were identified within the analyzed works and integrated into a validated competence model in the areas of technical, methodological, social, and personal competences. The measurement instruments mainly used self-assessment of skills and knowledge as an indicator of competence and differed greatly in their statistical quality.

**Conclusions:**

The identified multitude of subcompetences illustrates the complexity of digital competence in health care, and existing measuring instruments are not yet able to reflect this complexity.

## Introduction

### Background

The 2006 European Parliament recommendation on key competences for lifelong learning lists digital competences as 1 of the 8 key competences for every citizen to enable personal fulfillment, active citizenship, social cohesion, and employability in our modern society [1]. Therefore, it is no surprise that the digital transformation within the health care sector, involving new processes and technologies [2], has completely changed the demands on people working in health care professions. Digital competence in health care is needed [3,4]. According to Vitello et al [5], competence is “the ability to integrate and apply contextually-appropriate knowledge, skills and psychosocial factors (e.g., beliefs, attitudes, values and motivations) to consistently perform successfully within a specified domain.” Salman et al [6] divide competence into 2 aspects: hard and soft. The hard aspects of competence include knowledge, skill, and behavior, whereas the soft aspects include character traits, motives, attitudes, values, and self-image. Together, all these aspects determine the performance or output—both visible and invisible—of an individual in a particular job. *Competence*, in contrast to *competency*, is attached to the person rather than to a task or activity [5], which fits better within this work because we are focusing not on specific digital activities but on how professionals deal with digital technologies when working in the health care domain. This is why we concentrate on competence in this work.

The updated version of the digital competence framework for citizens (DigComp 2.2) [7] divides digital competences for private individuals into 5 main dimensions: information and data literacy, communication and collaboration, digital content and creation, safety, and problem-solving. Specific knowledge, skills, and attitudes are assigned to each of these dimensions. Along with the requirements for digital competence in private life, there are certain requirements to be met before one can be considered digitally competent in professional life in the health care sector.

Unfortunately, to date, there is no standard definition for the construct *digital competence* within the health care domain. Although the topic of interest is *digital competence*, the term *digital literacy* was also considered because this term is more common in English-speaking countries, and both concepts are often used synonymously [8]. Currently, for both terms, different understandings exist [9]. In this review, the semantic meaning of the terms is important, that is, *the skills and characteristics required to navigate the (professional) digital world*.

The lack of a uniform definition also leads to problems in determining digital competence for health professionals: authors criticize the lack of validated and up-to-date instruments to measure digital literacy or digital competence in this field [10,11]. With existing measurement tools, the focus is solely on technical skills; the related aspects that also affect the use of digital technologies are neglected [10].

Therefore, the objective of this research was to create an overview of how digital competence is defined and measured among health care professionals and thus to provide a holistic picture.

### Research Questions

Primarily, the following questions will be answered with the help of the literature review:

- What definitions exist of the digital competence of health care professionals?
  - What are the similarities and differences among the various definitions?
  - On which basic models are the different definitions based?
- What possibilities exist for measuring the digital competence of health care professionals?
  - Which dimensions of digital competence are measured?
  - How are the dimensions measured (self-assessment, performance tasks, etc)?
  - Have the assessment tools been validated? What quality criteria have been applied?

## Methods

### Overview

To provide a systematic overview of existing research literature on digital literacy in health professions, we conducted a scoping review [12]. The review follows the JBI methodology for scoping reviews [13] (based on the works of Arksey and O’Malley [14] and Levac et al [15]), which follows these steps: (1) defining and aligning the objectives and questions; (2) developing and aligning the inclusion criteria with the objectives and questions; (3) describing the planned approach to evidence searching, selection, data extraction, and presentation of the evidence; (4) searching for the evidence; (5) selecting the evidence; (6) analysis of the evidence; (7) presentation of the results; and (8) summarizing the evidence in relation to the purpose of the review, making conclusions, and noting any implications of the findings.

The review was planned beforehand by AM and SM, including choosing the review method, formulating the research questions, selecting the databases, phrasing the search terms, and determining the eligibility criteria. AM screened the search results, during which process there was regular professional exchange with another author, VW. The results were reviewed by SM, VW, and JN. AM, SM, VW, and JN all have experience in conducting scoping reviews.

To ensure the high quality and informative value of the results report, the description of the results is based on the PRISMA-ScR (Preferred Reporting Items for Systematic Reviews and Meta-Analyses extension for Scoping Reviews) checklist [12,16] (Multimedia Appendix 1). In addition, an evaluation protocol was prepared in advance of the review and made publicly available on OSF [17].

### Search Strategy

The literature search took place in April 2023 and used the databases ScienceDirect, Scopus, PubMed, EBSCOhost (which provides results from MEDLINE, OpenAIRE, ERIC, and OAIster), Cochrane Library, CAMbase, APA PsycNet, and Psyndex. The search term used was as follows: (“digital competence” OR “digital literacy”) AND (“medical professional” OR “healthcare professional” OR “healthcare worker” OR “physician assistant” OR “health professional”).

Fixed combinations of terms (such as digital literacy) are placed in quotation marks. Parentheses are used to force the right evaluation order of the expression. No adjacent terms were added so as not to make assumptions about the nature of the terms of interest. These were combined with various health worker designations. Neutral terms were chosen for the designation of nonmedical personnel to achieve a neutral and comprehensive understanding for different health professions. The keywords were linked with the Boolean operator “OR” to show results with at least one of the given terms. The operator “AND” ensures that all search results contain both “digital competence” or “digital literacy” and a health worker designation. The search term was developed through several trial cycles of a combination of terms. These were entered into the different databases and, based on the search results, terms were added or removed. The results are shown in Table 1.

**Table 1:** Results of the database search. The search term (“digital competence” OR “digital literacy”) AND (“medical professional” OR “healthcare professional” OR “healthcare worker” OR “physician assistant” OR “health professional”) was used for each database (N=1682).

Database	Results, n (%)
ScienceDirect	594 (35.31)
Scopus	361 (21.46)
PubMed	15 (0.89)
EBSCOhost (MEDLINE, OpenAIRE, ERIC, and OAIster)	706 (41.97)
Cochrane Library	6 (0.36)
CAMbase	0 (0)
APA PsycNet	0 (0)
Psyndex	0 (0)

### Eligibility Criteria

This scoping review considered peer-reviewed publications that were research articles, book chapters, review articles, or conference papers published within the last 10 years (2013-2023). Papers in either English or German were included.

The articles address the digital competence of health care workers in both outpatient and inpatient care. They come from medical, technical, or educational research fields. Papers from the patient’s perspective or those that address eHealth literacy or digital health literacy, defined as the “skills, knowledge and resources to search for, find, understand, evaluate and apply health information [from the internet]” [18], were excluded because the concept of interest is more concerned with the understanding of information rather than with the professional use of digital technologies. The overall eligibility criteria for this scoping review are presented in Textbox 1.

**Inclusion and exclusion criteria for the scoping review.**

**Inclusion criteria**

- Peer-reviewed publications
- Research articles, book chapters, review articles, or conference papers
- Research field: medical, technical, or educational
- Subject: articles addressing digital competence or digital literacy
- Population: health care workers in both outpatient and inpatient care and students and graduates of health care professions
- Period: articles published from 2013 to 2023
- Language: English or German

**Exclusion criteria**

- Not peer-reviewed publications
- Research field: any research field other than medical, technical, or educational
- Subject: articles addressing eHealth literacy or digital health literacy
- Population: patients
- Period: articles published before 2013
- Language: other than English or German

### Article Screening and Data Extraction

According to the recommendations of Moher et al [19], these steps are followed in the study selection process: first, duplicates are removed from the initial search results, after which the remaining publications are evaluated based on their titles, keywords, and abstracts and, subsequently, checked for suitability based on the full texts. The eligible papers are included in the review [19]. We followed the recommended process and, from the eligible papers, extracted and listed the following data in a Microsoft Excel sheet that was developed a priori but refined iteratively: authors, year of publication, country of origin, type of survey, and target group.

### Synthesis of Results

We present the characteristics of the selected studies, with a comparison of the drafted definitions of digital competence. In addition, we report the fundamental frameworks, models, and research papers that originally specified these definitions. We have collected and clustered all competences mentioned in the eligible papers. The structuring of the competences identified in the works was based on the competence categories according to the competence model developed by Hecklau et al [20], who cluster competences into technical, methodological, social, and personal competences to achieve clarity and transparency of the competence model. This clustering was adopted within our work to organize the determined competences. Finally, we explicitly examine the papers in which digital literacy assessment tools are used, with a consideration of the origin of the questionnaires, the form of measurement, and an assessment of their statistical quality.

## Results

### Selection of Sources of Evidence

**Figure 1:**
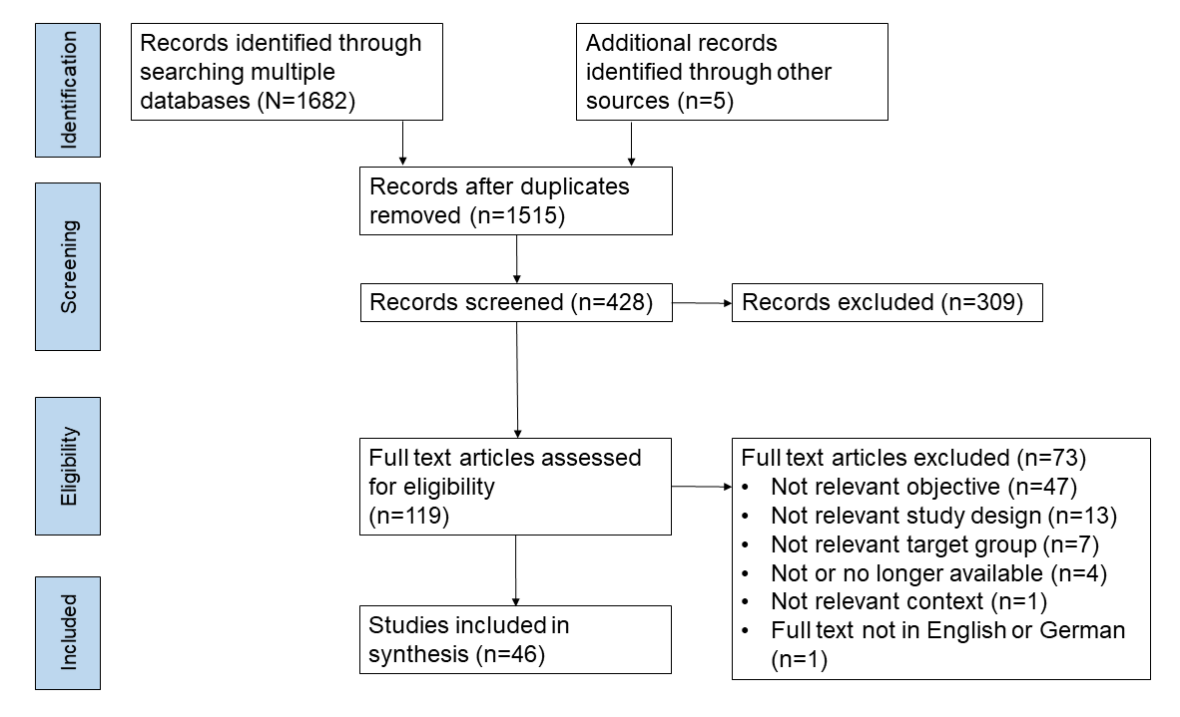
PRISMA (Preferred Reporting Items for Systematic Reviews and Meta-Analyses) flowchart showing the number of articles identified, screened, assessed for eligibility, and included in the final analysis.

The initial search identified 1682 papers (Table 1), of which 1510 (89.77%) remained after duplicates were removed. After applying the inclusion criteria (time period, type, and language) and screening the titles, of the 1510 papers, 428 (28.34%) were available for preselection, which, after the screening of the abstracts, reduced to 119 (27.8%) titles. Finally, after consideration of the full texts, of the 1682 papers identified through the initial search, 46 (2.73%) were included in this scoping review (Figure 1).

### Characteristics of Sources of Evidence

The selected papers were largely published from 2020 onward (35/46, 76%), indicating an increase in the perceived relevance of digital literacy among health professionals in the scientific world. In the years prior (2013-2019), only 11 (24%) of the 46 papers were published, with a slightly perceptible increase from 1 (9%) paper in 2014 to 4 (36%) in 2019. Of the 46 papers, the maximum number was published in 2020 (n=15, 33%); in subsequent years, the number of papers decreased to 8 (17%) in 2021 and 6 (13%) in 2022, and in 2023, a total of 6 (13%) papers had been published until May of that year. Table 2 shows the key data of the included papers.

**Table 2:** Key data of the included papers.

Authors	Year	Country	Type of study	Target group
Awami [21]	2020	Libya	Quantitative study	Health care professionals
Barbosa et al [22]	2023	Austria, Belgium, Croatia, Denmark, Finland, France, Italy, Malta, Netherlands, Norway, Poland, Portugal, and United Kingdom	Quantitative study	Radiotherapists
Brice and Almond [23]	2020	Australia	Scoping review	Health care professionals
Brown et al [24]	2020	Australia	Quantitative study	Nurses
Burzynska et al [25]	2023	Poland	Quantitative study	Physicians
Butler-Henderson et al [26]	2020	Australia	Meta-analysis	Health care professionals
Cabero-Almenara et al [27]	2021	Spain	Quantitative study	Health science lecturers
Cham et al [28]	2022	Australia	Quantitative study	Students of health professions
Coldwell-Neilson et al [9]	2019	Australia	Framework development	Optometry students
Evangelinos and Holley [29]	2014	United Kingdom	Qualitative interview	Health care professionals
Faihs et al [30]	2022	Germany	Quantitative study	Medical students
Golz et al [31]	2021	Switzerland	Quantitative study	Health care professionals
Hallit et al [32]	2020	Lebanon	Quantitative study	Pharmacists
Hilty et al [33]	2021	United States	Scoping review	Health care professionals
Holt et al [34]	2020	Denmark	Quantitative study	Nursing students
Jarva et al [35]	2022	Finland	Qualitative interview	Health care professionals
Jarva et al [36]	2023	Finland	Questionnaire development	Health care professionals
Jimenez et al [37]	2020	Singapore	Scoping review	Health care professionals
Jose et al [38]	2023	Chile	Scoping review	Health care professionals
Kaihlanen et al [39]	2021	Finland	Quantitative study	Nurses
Kayser et al [40]	2022	Denmark	Quantitative study	Health care professionals
Kim and Jeon [41]	2020	South Korea	Quantitative study	Nursing students
Konttila et al [42]	2019	Finland	Systematic review	Health care professionals
Kuek and Hakkennes [11]	2020	Australia	Quantitative study	Health care professionals
Longhini et al [10]	2022	Italy	Systematic review	Health care professionals
MacLure and Steward [43]	2018	United Kingdom	Qualitative interview	Pharmacists
MacLure and Steward [44]	2016	United Kingdom	Systematic review	Pharmacists
Matthews [45]	2021	United Kingdom	Systematic review	Health graduates
McGregor et al [46]	2017	Australia	Qualitative interview	Health graduates
Montebello et al [47]	2016	Malta	SWOT^a^ analysis	Students of health professions
Nazeha et al [4]	2020	Singapore	Scoping review	Health care professionals
O’Connor and LaRue [48]	2021	United Kingdom	Framework development	Nurses
Oo et al [49]	2021	Myanmar	Quantitative study	Health care professionals
Poncette et al [50]	2020	Germany	Mixed methods study	Medical students
Pontefract and Wilson [51]	2019	United Kingdom	Qualitative interview	Health care professionals
Rachmani et al [52]	2020	Indonesia	Quantitative study	Health care professionals
Reixach et al [53]	2022	Spain	Quantitative study	Health care professionals
Shiferaw et al [54]	2020	Ethiopia	Quantitative study	Health care professionals
Skiba et al [55]	2017	United States	Historical development report	Health care professionals
Tegegne et al [56]	2023	Ethiopia	Quantitative study	Health care professionals
Värri et al [57]	2020	Finland	Framework development	Students of health professions
Vehko et al [58]	2019	Finland	Quantitative study	Nurses
Virtanen et al [59]	2021	Finland	Systematic review	Health care professionals
Vissers et al [60]	2018	International	Quantitative study	Physiotherapy students
Whittaker et al [61]	2020	New Zealand	Quantitative study	Health care professionals
Wubante et al [62]	2023	Ethiopia	Quantitative study	Health care professionals

^a^SWOT: strengths, weaknesses, opportunities, and threats.

The majority of the articles were published in Australia (7/46, 15%), Finland (7/46, 15%), and the United Kingdom (6/46, 13%). The remaining papers were distributed worldwide: Ethiopia (3/46, 7%); Denmark, Germany, Singapore, Spain, and the United States (2/46, 4% each); and Chile, Indonesia, Italy, Lebanon, Libya, Malta, Myanmar, New Zealand, Poland, South Korea, Switzerland, 13 countries in Europe (Austria, Belgium, Croatia, Denmark, Finland, France, Italy, Malta, Netherlands, Norway, Poland, Portugal, and the United Kingdom), and the rest of the world (1/46, 2% each).

The types of papers were mainly distributed between quantitative studies (23/46, 50%) and reviews (scoping reviews, systematic reviews, and meta-analyses; 11/46, 24%). Less represented were qualitative interviews (5/46, 11%) and framework development (3/46, 7%), as well as questionnaire development; mixed methods study; strengths, weaknesses, opportunities, and threats analysis; and historical development report (1/46, 2% each).

The papers’ target group was largely unspecific, with most of them addressing *health care professionals* (25/46, 54%). Other papers addressed specifically *nurses* (4/46, 9%), *pharmacists* (3/46, 7%), *health graduates* (2/46, 4%), *health science lecturers* (1/46, 2%), *physicians* (1/46, 2%), and *radiotherapists* (1/46, 2%). Some of the papers were aimed at students: students of health professions in general (3/46, 7%), medical students and nursing students (2/46, 4% each), and optometry students and physiotherapy students (1/46, 2% each).

### Definition of Data Literacy

The main difficulty concerning the literature analysis was that some of the papers used the term *digital literacy* but actually referred to a different concept (especially *eHealth literacy*). When selecting the papers for review, articles that dealt, in terms of semantics, with concepts other than *data literacy* were sorted out.

Most of the papers provided definitions in which digital competence is composed of various dimensions of competence. There was a strong focus on skills in the formulated definitions of digital competence [9,21,22,25-33,35,36,39,40,42,43,45, 47,48,50-55,59,60,62]. Many papers (27/46, 59%) also stated in their definitions that certain kinds of knowledge are necessary for competence [4,10,22,23,25-28,30-33,36,39,40,42,47, 49-55,59,60,62]. Some of the papers (17/46, 37%) proposed that the attitude toward technical issues should be considered a component of competence [4,10,11,21,24,27,28,30-33,36,42, 47,49,54,59]. Other papers (6/46, 13%) added that former experiences with digital topics play a crucial role in forming competence [28,31,40,42,43,46]. According to Konttila et al [42], experiences are the base for the emergence of attitudes. Other works mentioned motivation (7/46, 15%) [31,35,36,40,42,57,59], practices (2/46, 4%) [9,31], consciousness (2/46, 4%) [9,54], fears (2/46, 4%) [11,43], goals (1/46, 2%) [25], identity (1/46, 2%) [9], self-awareness (1/46, 2%) [28], and strategies (1/46, 2%) [54] as part of competence. These competence dimensions provide a framework for the required competence areas, which are described in the *Identified Competence Areas and Competences* subsection.

The definitions used are either the results of scoping reviews or frameworks where many individual results have been merged (15/46, 33%) [4,10,23,26,28,33,37,42,44,46,48,51,52,59,61]. Alternatively, they are based on other, explicitly named works, such as DigComp 2.2 [7] (4/46, 9%) [22,29,54,56]; the European framework for the digital competence of educators [63] (1/46, 2%) [27]; the technology acceptance model [64] and the unified theory of acceptance and use of technology [65] (1/46, 2%) [11]; the accreditation of competence in information and communication technologies by the government of Catalonia [66] (1/46, 2%) [53]; the Educause Center for Analysis and Research [67] (1/46, 2%) [60]; the General Confidence with Computer Use Scale [68] (1/46, 2%) [32]; the eHealth literacy questionnaire [69] (1/46, 2%) [40]; the eHealth literacy assessment toolkit [70] (1/46, 2%) [34]; the Self-Assessment of Nursing Informatics Competencies Scale [71] (1/46, 2%) [24]; a scale assessing the informatics competencies for nurses [72] (1/46, 2%) [39]; a scale assessing digital literacy with regard to information and communication technology [73] (1/46, 2%) [41]; the definition by Konttila et al [42] (1/46, 2%) [31]; the definition by Ferrari [74] (1/46, 2%) [21]; the definition by Bawden [75] (1/46, 2%) [25]; the definition by Sharpe and Beetham [76] (1/46, 2%) [9]; the definition by Hecklau et al [20] (1/46, 2%) [38]; the definition by Gretton and Honeymen [77] (2/46, 4%) [43,44]; the Health Education England definition [78] (1/46, 2%) [45]; the Jisc 7 elements of digital literacies (1/46, 2%) [47]; the World Health Organization’s *Electronic Health Records: A Manual For Developing Countries* [79] (1/46, 2%) [49]; and the definition by Skiba et al [80] (1/46, 2%) [57]. No information was provided in 4 (9%) of the 46 studies [30,50,58,62] about the basis of the definition used. Montebello et al [47] refers to the Jisc 7 elements of digital literacies as basis for their digital literacy definition but the original source is not available anymore.

### Identified Competence Areas and Competences

#### Overview

Within the included papers, competences in the 4 main competence areas according to the model developed by Hecklau et al [20] were identified: multiple competences could be grouped into technical, methodological, social, and personal competences. All these competences, classified into 4 competence areas, are described in the following paragraphs and depicted in Textbox 2.

**The identified competences grouped into different competence areas.**

**Competence areas and competences**

- Technical competences
- Basic computer competence [4,9,11,21-25,27-29,32,33,35-39,41,43-45,47-49,51-54,56,57,62]
- Basic competence to use wireless devices [21,23-25,37,49]
- Applied digital health skills [4,10,22,24,26,29,30,33,35,37,39,40,42,43,46,48,50-53,55,57,58,61,62]
- Anticipation of advanced and future digital competences [30,37,38,41,48,50,57]
- Administration of technology [4,23,45]
- Ethical aspects of digitalization [4,36,37,48,50,57,58]
- Legal aspects of digitalization [4,37,48,50,52]
- Methodological competences
- Data and information processing competence [4,9,21,22,24-26,29-31,35,37,38,40,41,44,45,47,48,50-57,62]
- Continuous learning [4,9,23,25,28-30,32,38,41,45-47,49,54,55,57,62]
- Project management [4,57,61]
- Research competence [4,37,45,47,57]
- Problem-solving [22,35,38,41,54,56,62]
- Social competences
- Working in teams [9,23,29,35,38,41,42,45,47,50,51,53-55,62]
- Communication competence [4,9,22,29-31,35,36,38,42,43,45,47,49-51,54-57,59,62]
- Networking skills [38,47,50]
- Teaching [27,45]
- Focus on patients [4,10,35-37,48,50,55,57]
- Personal competences
- Innovative behavior [23,38,45,50]
- Self-reflection [35,53,54]
- Critical thinking [22,25,54]
- Creativity [38,54]
- Professionalism [23]

#### Technical Competences

Multiple subcompetences of technical competences were identified: the ones mentioned most often were *basic computer competence*, meaning knowledge of different computer components and basic computer concepts [21,32,43]; and skills in using hardware (eg, switching equipment on and off and operating input and output devices) [49,62]. Internet use, consisting of navigating the internet, knowledge of various internet sources, and finding and downloading articles, is part of basic computer competence [24,25,28,37,43,52,62]. The users should be able to use and install software [24,28,32,33,37,49,52,62] and especially be able to use information and communication technology, including understanding the basic concepts and components of information and communication technology and designing, creating, integrating, publishing, and revising content [4,9,22,23,27,35-38,41,43-45,47-49,53,54,56,57,62]. Another part of basic computer competence is file management and comprehensive knowledge of file formats, the creation of documents and folder structure [37,49], and IT security (eg, using passwords and antivirus tools) [22,29,37,38,45,52,54,56,62].

Another subcompetence mentioned was *basic competence to use wireless devices*, consisting of operating hardware [49], using the internet [21,37], managing files [21,37], and using applications [21,37].

Existing competences can be transferred to eHealth contexts to achieve the foundation for *applied digital health skills* [46]. Here, one of the largest areas is the use of health applications, meaning the use of various digital health solutions for treatment planning, diagnostics, treatment, processing imaging data, and so on [22,24,33,35,40,42,48,57,58]. This includes the management of electronic patient records [22,24,37,43,49,51,57,58,62], the use of wearables and mobile health apps [30,57], the administration of electronic documentation [4,37], and the use of health information systems [37,52,55,57]. In addition, health professionals need skills and knowledge about specific data protection and security requirements of their profession [4,30,48,53]. Furthermore, digitally competent health care workers need to be able to establish new technologies in their work environments and participate in the design, implementation, and evaluation of systems, as well as seek available resources, formulate ethical decisions technical wise, and promote the use of IT in health environments [4,24,42,48,50,57].

A further subcompetence is the *anticipation of advanced and future digital competences,* where users stay informed about the current state of the art of digital technologies and the competences that are necessary to use these [38,41], as well as how certain technologies will develop in the future, which play a role in the future of health care (eg, big data, artificial intelligence, robotics, and genomics) [30,37,48,50].

One crucial aspect of technical competence is the *administration of technology*, which encompasses planning, implementation, optimization, and operation or management, as well as the control of technological products or tools, processes, and services [4,23,45].

Knowledge about *ethical aspects* [4,36,37,48,50,57,58], such as freedom of choice, privacy, autonomy, and fairness [36], as well as the *legal aspects of digitalization* [4,37,48,50,52,62], in particular regarding the regulation of medical practice and medical devices [50] and the protection of patient data as well as confidentiality when processing data [52], is equally important when handling new technologies to enable data protection and data security.

#### Methodological Competences

The competence to *process data and information* consists of finding [4,23,24,26,37,44,47,52,53,62], evaluating [21,23,25,37,43,47,50-53,57,62], creating [23,24,44,49,51], managing [4,23,24,26,29,30,47-49,52,53,57], sharing or communicating [4,23,26,31,44,47,53,57], analyzing [4,26,37,50,53], visualizing [4], and interpreting [24,26,47,49] information or data; deriving actions or decisions [50]; being well versed in data protection and security [50,51]; and knowing the difference among data, information, and knowledge [48].

In addition, the ability to *continuously learn* is a fundamental component of digital competence. Learning is described as using educational methods such as teaching, training, storytelling, discussion, and targeted research to acquire knowledge, skills, values, beliefs, and habits [23]. It includes the anticipation of service and training needs and, for future digital literacy skills [57], learning how to use new technologies [29,49,62] and acquiring new concepts, methods, and tools [23], especially by using digital teaching and learning resources [4,29,41,47].

Digitally competent health professionals should also be proficient in *project management* to be able to introduce new operating models and lead IT-based change in their field [4,57,61].

They should be able to use IT for research support and innovations [4] as well as for assessment and continuous improvement of their own skills, their work community skills development, and the development of electronic services [57] through *research competence*.

*Problem-solving* competence can be interpreted as both dealing with digital problems [22,35,38,54,56] and solving problems through digital means [41,54,56,62].

#### Social Competences

To engage digitally in the social work environment, digitally competent health professionals must be able to *work in teams*, meaning they should be able to work cooperatively or collaboratively [9,23,38,41,45,47,50,53,62]; take a leadership role [38]; deal with diverse teams consisting of members with different demographics, from different professions, and with different personality traits [38,51]; be willing to compromise for the sake of group harmony [38]; and establish collegial support to create positive digital experiences [35,42].

Another basic requirement to work in (digital) teams is *communication competence* using a wide range of communication methods [50], including digital communication [4,9,30,38,57,62] (eg, web-based meetings and consultations and the use of social media [57] within the team [36,57] and with patients [35,36]). Digitally competent health professionals need to know the correct vocabulary [57] and, with this knowledge, the ability to share knowledge [38].

*Networking skills* are evident in the use of knowledge networks, where health professionals participate in digital networks for learning and research and develop an open-access mentality [38,47,50].

Health professionals should not only be able to gain knowledge but also to pass it on: *teaching* is an important part of digital literacy. Health professionals could impart their knowledge using digital resources and provide these resources to learners, assess their learning success, and increase not only their own but also the learners’ digital literacy [27,45].

Another important part of digital literacy is keeping the *focus on patients* by considering the patients’ digital needs and evaluating their digital skills, as well as considering their willingness to use digital services to provide services that they feel safe to use and capable of using [35,57]. In addition, health professionals should promote the use of IT among patients through support and empowerment for self-management, IT guidance (eg, guides and web-based materials), and support in finding information [4,57].

#### Personal Competences

To be digitally competent, health professionals need *innovative behavior* as a personality trait, meaning they should have the spirit of invention and lifelong determination [23,38,45,50]. The initiative to conceive, consider, try out, or apply new ideas, products, processes, and procedures to their individual work role or their work unit without fear of change [23] is essential to drive the transformation process of health care forward [50].

Another relevant ability for health professionals is *self-reflection* with regard to their own digital competence [35,53,54] and the identification of personal and professional needs to apply technical solutions [53].

Other personal traits mentioned as relevant for digital competence are *critical thinking* [22,25,54] and *creativity* [38,54]. Critical thinking is mentioned in connection with information evaluation [25] or gaining new information within a professional context [22,54]. Creativity is of use when knowledge is built up [54] or a task has to be approached with an innovative mindset [38].

*Professionalism* is defined as the behavior, demeanor, and attitude of a person in a work environment and is considered a useful quality rather than a requirement of a role [23], but it is a characteristic that is beneficial to health professionals wishing to be digitally competent.

### Measurement Instruments

Of the 46 included papers, 25 (54%) used different questionnaires to evaluate the digital literacy of health professionals. The majority of the questionnaires used (15/25, 60%) [21,22,25,28,30-32,36,49,50,52,53,58,61,62] were developed originally for these papers. Others used existing questionnaires or frameworks (Textbox 3) such as the Self-Assessment of Nursing Informatics Competencies Scale [71] in the study by Brown et al [24]; a scale assessing the informatics competencies for nurses [72] in the study by Kaihlanen et al [39]; the eHealth literacy assessment toolkit [70] in the study by Holt et al [34]; the eHealth literacy questionnaire [69] in the study by Kayser et al [40]; the General Confidence with Computer Use Scale [68] in the study by Hallit et al [32]; the attitudes and digital literacy toward information and communication technology scale [73] in the study by Kim and Yeon [41]; the Educause Center for Analysis and Research [67] in the study by Vissers et al [60]; the technology acceptance model [64] and the unified theory of acceptance and use of technology [65] in the study by Kuek and Hakkennes [11]; DigComp 2.2 [7] in the studies by Barbosa et al [22], Shiferaw et al [54], and Tegegne et al [56]; the European framework for the digital competence of educators [63] in the study by Cabero-Almenara et al [27]; and the accreditation of competence in information and communication technologies by the government of Catalonia [66] in the study by Reixach et al [53].

**Underlying work for the questionnaires used in the studies.**

**Underlying work and corresponding studies**

- Technology acceptance model [64] and unified theory of acceptance and use of technology [65]
- Kuek and Hakkennes [11]
- Updated version of the digital competence framework for citizens [7]
- Barbosa et al [22], Shiferaw et al [54], and Tegegne et al [56]
- Self-Assessment of Nursing Informatics Competencies Scale [71]
- Brown et al [24]
- Informatics competencies scale for nurses [72]
- Kaihlanen et al [39]
- eHealth literacy assessment toolkit [70]
- Holt et al [34]
- eHealth literacy questionnaire [69]
- Kayser et al [40]
- General Confidence with Computer Use Scale [68]
- Hallit et al [32]
- Attitudes and digital literacy toward information and communication technology scale [73]
- Kim and Yeon [41]
- Educause Center for Analysis and Research [67]
- Vissers et al [60]
- European framework for the digital competence of educators [63]
- Cabero-Almenara et al [27]
- Accreditation of competence in information and communication technologies by the government of Catalonia [66]
- Reixach et al [53]

Digital literacy was measured in various forms, and some questionnaires used different combinations of measurement forms (Textbox 4). The specific items of the questionnaires considered in the review are categorized thematically herein. In many surveys, participants provided a self-assessment of specific skills and knowledge. Often, they had to assign certain abilities or confidence levels to themselves [11,22,24,25,27,28,30-32,34,36,39-41,49,52-54,56,58,61,62]. Other questionnaires collected participants’ attitudes toward technical topics [11,21,24,30,31,36,40,41,50,62]. Some items dealt with the experiences or needs of participants with regard to (further) training in digital topics [21,25,30,49,50,53,56,62]. Another way of measuring digital literacy involved requesting access to different technologies, such as smartphones, laptop computers, or tablet devices, for private or professional use [28,32,49,60,62] or the frequency of use of these technologies [11,25,28,40,60]. Other items addressed user behavior: what the devices were used for [24,49,60], and which applications were used [21,24].

The questionnaires differed greatly in their statistical quality. Some have not been validated in any statistical form [21,25,28,39,50,58,60-62], whereas others were only tested on internal consistency [41,49,53], and several were verified with different reliability and validity tests [11,22,24,27,30-32,34,36,40,52,54,56].

**Different measurement forms of digital literacy with item examples.**

**Measurement form and item examples**

- Self-assessment [11,22,24,25,27,28,30-32,34,36,39-41,49,52-54,56,58,61,62]
- “I can use the most common computer programs and services (e.g. email, intranet) in my work.” [36]
- “How well do you feel you master the following skills required to use information systems?” [58]
- Attitudes [11,21,24,30,31,36,40,41,50,62]
- “I believe that new digital technologies will fundamentally change medicine in the next few years.” [30]
- “The transfer to digital services is a positive change.” [36]
- Experiences, needs of education, or training [21,25,30,49,50,53,56,62]
- “I would benefit from additional trainings/courses in the field of shaping digital competences.” [25]
- “On a personal level, would you like to have specific training in any of the following areas? eg. Digital culture, participation and citizenship using digital tools.” [53]
- Access to technology [28,32,49,60,62]
- “Do you think you have internet access in your office?” [62]
- “Owning a computer.” [32]
- Frequency of use [11,25,28,40,60]
- “Please state how often you use the following in your work and in your personal life: computers, Microsoft Office applications, smartphones, tablets, email, the internet, and social media (i.e. Facebook, Twitter and Instagram).” [11]
- “How often do you use the internet?” [60]
- User behavior [21,24,49,60]
- “I use MS Excel for work.” [21]
- “What is the purpose of [sic] you use a computer?: work, education, communication, entertainment, and playing games” [49]

## Discussion

### Principal Findings

The selected literature sources show the increasing scientific interest in digital literacy in health care and the worldwide spread of this development. There is a focus on quantitative research, although, because the available survey instruments were considered insufficient to determine digital literacy, researchers often developed their own. The underlying definitions are based on a variety of approaches and sources, which highlights the need for a structured overview. Most of the definitions focused on skills and knowledge as indicators of competence. *Soft aspects*, as described by Salman et al [6], were also mentioned by authors but less frequently and in many different forms. Attitude, experience, and motivation were mentioned most often. Behavior, which is a *hard aspect* according to Salman et al [6], was not addressed explicitly in the definitions provided in the included papers.

The identified competences have been categorized according to the competence categories formulated by Hecklau et al [20]. The determined technical competences include basic computer competence, basic competence to use wireless devices, applied digital health skills, anticipation of advanced and future digital competences, administration of technology, ethical aspects of digitalization, and legal aspects of digitalization. Data and information processing competence, continuous learning, project management, research competence, and problem-solving were mentioned in the literature as methodological competences. The following were classified as social competences: working in teams, communication competence, networking skills, teaching, and focus on patients. Personal competences include innovative behavior, self-reflection, critical thinking, creativity, and professionalism.

The results confirm that existing measurement tools focus solely on technical areas [10], and other related aspects, such as the identified competences from the methodological, social, and personal areas in other nonquantitative works, have not been taken into account. Unlike what Longhini et al [10] and Kuek and Hakkennes [11] stated, many of the questionnaires used had high statistical quality and were verified with different reliability and validity tests. The questionnaires largely measure digital literacy via self-assessment. Some also use items relating to attitudes, experiences, access to technology, frequency of use, and use behavior.

The allocation of competences to the categories was sometimes not trivial and not clearly distinguishable; for example, *teaching* could be categorized as both a social and a methodological competence. How the partial competence areas are connected also remains unanswered in these works. Hurst [81] describes 3 possible dependency relationships: a general factor model where basic competence is composed of equally important subaspects, an additive model where the individual subaspects have a juxtaposed relationship, or a hierarchical model where basic subcompetences and higher-level competences exist that build on each other [81]. A more complex consideration of the relationships among the individual competences, for example, through a factor analysis, would also be conceivable and should be investigated in subsequent research work. Some of the skills identified are specifically linked to digital topics, but others are more general and *analog* in nature, especially in the social and personal categories. Therefore, mutual influences among the competences are not only conceivable but also probable.

### Limitations

One limitation of this literature review is that, because of the very nature of scoping reviews, the quality of the included works was not considered in the review process, and all papers were included in the synthesis, irrespective of quality [14]. This may have led to inferior works being included in the results and being placed on an equal footing with high-quality works. When constructing the search term, no wildcards were used, which limited the search of potential fitting literature, which must be specified as a further limitation. In addition, more variants of the job title *medical professional* could have been used to maximize the search results. Another limitation could be the practical implementation of the selection of papers and their evaluation by just 1 author. Although the procedure was planned as a team, and the results were discussed extensively, the process was carried out by only 1 person.

### Future Directions

This literature review focuses solely on the terms *digital competence* and *digital literacy* and provides an overview of the use of these closely related terms. A larger literature review that includes other adjacent topics, such as *informatics competences*, or refers to specific digital activities in the health care sector, such as *telemedicine competences*, would heighten the credibility in terms of an overall semantic understanding of the concept of competence when dealing with all sorts of digital technologies. Within this work, which aimed at an understanding of the specifically named term *digital competence*, the addition of related concepts would not be possible without the development of an initial understanding of this concept, which the authors have developed in the course of this work.

A further enrichment of an in-depth analysis would be the addition of specific medical specialties. The aim of this work was the nonspecific and generalizable consideration of required digital skills in health care, but, of course, every profession has its individual (digital) requirements that are worth considering.

### Conclusions

The review shows that the interest in digital literacy as a research topic in health care is currently on the rise but that the understanding of this rather abstract term is widely divergent. A uniform definition and use of terms is needed. The existence of hard and soft aspects of competence, as described by Salman et al [6], was confirmed by many of the used definitions, but which of the identified aspects contribute to what extent needs to be investigated further. Furthermore, the multitude of subcompetences illustrates the complexity of digital competence that needs to be taken into account when developing a measurement instrument. Well-validated questionnaires exist, these focus solely on technical aspects. The competency model identified in this work can be used as a starting point for factor analysis of the identified competences or questionnaire development.

## Appendix

Multimedia Appendix 1 PRISMA-ScR (Preferred Reporting Items for Systematic Reviews and Meta-Analyses extension for Scoping Reviews) checklist.

## Abbreviations

DigComp 2.2: updated version of the digital competence framework for citizens
PRISMA-ScR: Preferred Reporting Items for Systematic Reviews and Meta-Analyses extension for Scoping Reviews

## Data Availability

The data sets generated and analyzed during this study are available from the corresponding author upon reasonable request.
